# Vitamin E Intake Modulates the Effect of Selenomethionine on Sexual Function and Depressive Symptoms in Reproductive-Age Women with Euthyroid Autoimmune Thyroiditis: A Pilot Study

**DOI:** 10.3390/antiox15050549

**Published:** 2026-04-26

**Authors:** Robert Krysiak, Karolina Kowalcze, Johannes Ott, Giovanni Cangelosi, Simona Zaami, Bogusław Okopień

**Affiliations:** 1 Department of Internal Medicine and Clinical Pharmacology, Medical University of Silesia, Medyków 18, 40-752 Katowice, Poland; bokopien@sum.edu.pl; 2 Department of Pediatrics in Bytom, Faculty of Health Sciences in Katowice, Medical University of Silesia, Stefana Batorego 15, 41-902 Bytom, Poland; kkowalcze@sum.edu.pl; 3 Department of Pathophysiology, Faculty of Medicine, Academy of Silesia, Rolna 43, 40-555 Katowice, Poland; 4 Clinical Division of Gynecologic Endocrinology and Reproductive Medicine, Department of Obstetrics and Gynecology, Medical University of Vienna, 1090 Vienna, Austria; johannes.ott@meduniwien.ac.at; 5 School of Pharmacy, Experimental Medicine and “Stefania Scuri” Public Health Department, University of Camerino, 62032 Camerino, Italy; giovanni01.cangelosi@unicam.it; 6 Department of Anatomical, Histological, Forensic and Orthopedic Sciences, Sapienza University of Rome, 00161 Rome, Italy; simona.zaami@uniroma1.it

**Keywords:** antioxidants, fat-soluble vitamins, hypothalamic–pituitary–thyroid axis, selenium, sexual function, thyroid autoimmunity, women’s health

## Abstract

Oxidative stress appears to be implicated in both the initiation and progression of autoimmune thyroiditis. Selenomethionine, which exhibits antioxidant properties, has been shown to reduce thyroid antibody titers in patients with autoimmune thyroiditis. Recent evidence suggests that vitamin E, a fat-soluble antioxidant, may protect against the development of autoimmune thyroiditis, and that its supplementation has been associated with improvements in female sexual function. The objective of the present pilot study was to determine whether vitamin E intake modulates the effects of selenomethionine on female sexual function and depressive symptoms in individuals with thyroid autoimmunity. The study enrolled three groups of reproductive-age women with euthyroid autoimmune thyroiditis, with 26 participants in each group. The groups were matched for age, thyroid peroxidase antibody titers, and TSH levels and differed according to vitamin E intake: adequate intake (group A), low intake (group B), and high intake (group C). All participants received selenomethionine supplementation (200 µg/day) for six months. Antibody titers and hormone levels were measured, and participants completed questionnaires assessing female sexual function (FSFI) and depressive symptoms (BDI-II). At baseline, no differences in biochemical outcomes were observed between the groups, except for testosterone levels. The study groups differed in sexual desire and arousal domain scores, which were higher in group A than in the other two groups. Total FSFI scores, the remaining FSFI domain scores, and BDI-II scores did not differ between groups at baseline. Across all groups, selenomethionine reduced thyroid peroxidase and thyroglobulin antibody titers and increased SPINA-GD and the ratio of free triiodothyronine to free thyroxine; however, the effects on antibody titers were most pronounced in group A. An increase in SPINA-GT and testosterone levels following selenomethionine supplementation was observed only in group A. In this group, selenomethionine also led to significant improvements in total FSFI scores and all individual domain scores. In contrast, in the remaining groups, the effects of supplementation were limited to increases in domain scores for lubrication, sexual satisfaction, and pain. A treatment-related reduction in total BDI-II scores was observed exclusively in women with adequate vitamin E intake. These findings suggest, for the first time, that dietary intake of a natural antioxidant may influence the effects of exogenous selenomethionine on sexual function and depressive symptoms in reproductive-age women with euthyroid autoimmune thyroiditis.

## 1. Introduction

Hashimoto’s thyroiditis, also referred to as autoimmune thyroiditis, is the foremost cause of hypothyroidism in regions with adequate iodine intake. It is the most common organ-specific autoimmune disorder and ranks among the most prevalent diseases in humans [1,2]. The condition disproportionately affects women, with a female-to-male ratio of 4–10:1, and is common in women of reproductive age [3]. Histologically, the disease is characterized by the infiltration of thyroid tissue with lymphocytes, progressive fibrosis, and the destruction of follicular cells [4]. Serologically, it is defined by the presence of thyroid-specific autoantibodies, particularly thyroid peroxidase antibodies (TPOAb) and thyroglobulin antibodies (TgAb) [2]. Even individuals with normal hypothalamic–pituitary–thyroid axis function may experience clinical symptoms, including fatigue, weight gain, cold intolerance, constipation, and low mood [5]. Furthermore, euthyroid Hashimoto’s thyroiditis may be followed by insulin resistance, metabolic syndrome, increased cardiovascular risk, and impaired cognitive function [6,7,8,9].

Proper sexual health is a fundamental aspect of women’s health and well-being [10]. To date, few studies have evaluated sexual functioning in women with autoimmune thyroiditis. More than a decade ago, Oppo et al. [11] were the first to report that women of reproductive age with euthyroid autoimmune thyroiditis exhibited reduced libido, although sexual dysfunction in this population was less pronounced than in women with untreated hypothyroidism. In a subsequent study, which involved young women with various thyroid disorders, we found that those with euthyroid Hashimoto’s disease exhibited lower scores in sexual desire, lubrication, and sexual satisfaction compared with women without thyroid pathology [12]. Moreover, when hypothyroidism was present, sexual dysfunction was more pronounced if the underlying cause was autoimmune in nature than when it resulted from other etiologies, such as prior thyroidectomy, congenital absence or underdevelopment of the thyroid gland, or inherited defects in thyroid hormone synthesis [12]. These findings were consistent with those reported later by Bortun et al. [13], who observed poorer sexual functioning in young women with autoimmune thyroiditis compared with an age- and body mass index-matched healthy control group. The severity of sexual dysfunction correlated with the activity of the hypothalamic–pituitary–thyroid axis, being most pronounced in cases of clinically overt hypothyroidism and least pronounced in individuals in whom the autoimmune process had not yet resulted in thyroid hypofunction [13]. The only distinction between the last two studies [12,13] was that decreased mood was reported in euthyroid women with Hashimoto’s disease solely in the study by Krysiak et al. [12], which may reflect a greater degree of thyroid gland destruction, as inclusion criteria required, in addition to the presence of thyroid antibodies, the characteristic ultrasonographic pattern of the disease.

The risk of progression to hypothyroidism, the frequent clinical manifestation of the disease, and the potential for metabolic, cardiovascular, and neurological complications support the rationale for initiating treatment of autoimmune thyroiditis at a stage when hypothalamic–pituitary–thyroid axis activity remains within normal limits. Although levothyroxine therapy in this patient population may lead to subjective improvement in some individuals, it has been shown to reduce thyroid antibody titers only in some studies and is associated with a risk of iatrogenic thyrotoxicosis that is higher than in patients with overt hypothyroidism [14,15]. Therefore, a promising therapeutic approach in patients with euthyroid autoimmune thyroiditis is non-hormonal treatment, particularly the administration of vitamin D, selenium compounds, and myo-inositol [16]. The effectiveness of the first two options has been confirmed by the results of recently published meta-analyses [17,18,19]. In turn, a meta-analysis by Peng et al. [20] demonstrated greater efficacy of selenium compounds compared with vitamin D. All of the above compounds, particularly selenium and myo-inositol, exhibit significant antioxidant activity, and their beneficial effects may be attributable to the role of oxidative stress in the development and progression of Hashimoto’s thyroiditis [21,22].

Vitamin E, one of the four fat-soluble vitamins, is a potent antioxidant that regulates the production of reactive oxygen and nitrogen species, protects polyunsaturated fatty acids in cell membranes from oxidative damage, and modulates signal transduction pathways [23,24]. Higher baseline serum vitamin E levels have been associated with a reduced risk of all-cause mortality, as well as decreased mortality from cardiovascular disease, heart disease, stroke, cancer, and respiratory diseases [25]. Owing to its immunomodulatory properties, vitamin E may also confer protection against the development of various autoimmune disorders [26]. However, the association between vitamin E and autoimmune thyroiditis has been investigated in only a limited number of studies, which have yielded inconsistent results. Liu et al. [27] reported that higher vitamin E intake among men was associated with a lower prevalence of autoimmune thyroiditis. Similarly, Buczyńska et al. [28] observed negative correlations between thyroid antibody titers and dietary vitamin E intake. In contrast, Wang et al. [29] found no significant association between vitamin E intake and the risk of developing Hashimoto’s thyroiditis.

Recent evidence also suggests that vitamin E may play a role in the regulation of women’s sexual health. The activity of arylesterase, an enzyme with antioxidant properties, was found to be lower in women with diabetes-induced sexual dysfunction than in diabetic women without this complication [30]. In a six-week double-blind randomized clinical trial, supplementation with vitamin E (100 IU daily) in combination with ginseng was superior to placebo in improving sexual desire and satisfaction among young women with sexual dysfunction, as assessed using the Female Sexual Function Index (FSFI) questionnaire [31]. Furthermore, an eight-week triple-blind randomized controlled trial demonstrated that combined supplementation with saffron and vitamin E (50 mg daily) was more effective in improving overall sexual function and its specific domains than vitamin E alone in women of reproductive age with sexual dysfunction [32]. In a 12-week single-blind randomized clinical trial involving postmenopausal women with genitourinary syndrome of menopause, vaginal administration of vitamin E (100 IU) significantly improved libido, arousal, lubrication, and orgasm, with effects comparable to those of vaginal conjugated estrogen cream (0.625 mg) [33].

The impact of the treatment of autoimmune thyroiditis on women’s sexual health remains poorly understood. Evidence supporting the efficacy of levothyroxine is limited to patients with a distinct immune-mediated thyroid disorder—postpartum thyroiditis—in whom coexisting hypothyroidism was a prerequisite for study inclusion [34]. In contrast, in patients with Hashimoto’s disease presenting with euthyroidism, beneficial effects have been observed following the use of selenomethionine, myo-inositol, and vitamin D. However, vitamin D was superior to the remaining treatment options in improving female sexual response [35]. It is difficult to explain the reason for this finding, which contrasts with the results of a meta-analysis by Peng et al. [20]. This discrepancy may be explained by the high prevalence of vitamin D deficiency or insufficiency among the study participants [35]. However, the Polish population is also characterized by low selenium intake [36]. Low selenium intake is often accompanied by low vitamin E intake [37]. Thus, the current study aimed to examine whether vitamin E status determines the effect of selenomethionine on sexual function and depressive symptoms in women of reproductive age with euthyroid autoimmune thyroiditis.

## 2. Materials and Methods

The institutional review board approved the study protocol, and the research was carried out in compliance with the Declaration of Helsinki. All participants provided written informed consent after being informed of the study’s purpose, significance, and potential risks and benefits.

### 2.1. Study Population

This prospective, matched outpatient cohort study enrolled women of reproductive age (18–48 years) with recently diagnosed, previously untreated euthyroid autoimmune thyroiditis. Eligibility criteria included: (a) TPOAb titers greater than 100 U/mL; (b) serum thyroid-stimulating hormone (TSH) levels between 0.4 and 4.5 mIU/L; (c) free thyroxine concentrations of 10.1–21.5 pmol/L; (d) free triiodothyronine concentrations of 2.3–6.5 pmol/L; (e) serum 25-hydroxyvitamin D (25OHD) levels of 75–150 nmol/L; and (f) a hypoechoic thyroid echotexture with small hypoechoic nodules and/or surrounding echogenic septa on ultrasound examination. To minimize the impact of potential seasonal variations in the outcome measures, recruitment was conducted year-round, with approximately equal numbers of participants enrolled in each season.

Participants were excluded if they had positive antibodies against the TSH receptor, other endocrine or autoimmune disorders, cardiovascular, renal, or hepatic disease, other severe medical conditions, pregnancy or lactation, menopause, congenital or acquired reproductive system anomalies, a history of urogynecological surgery potentially affecting sexual function, or ongoing pharmacotherapy, including hormonal contraception.

**Figure 1:**
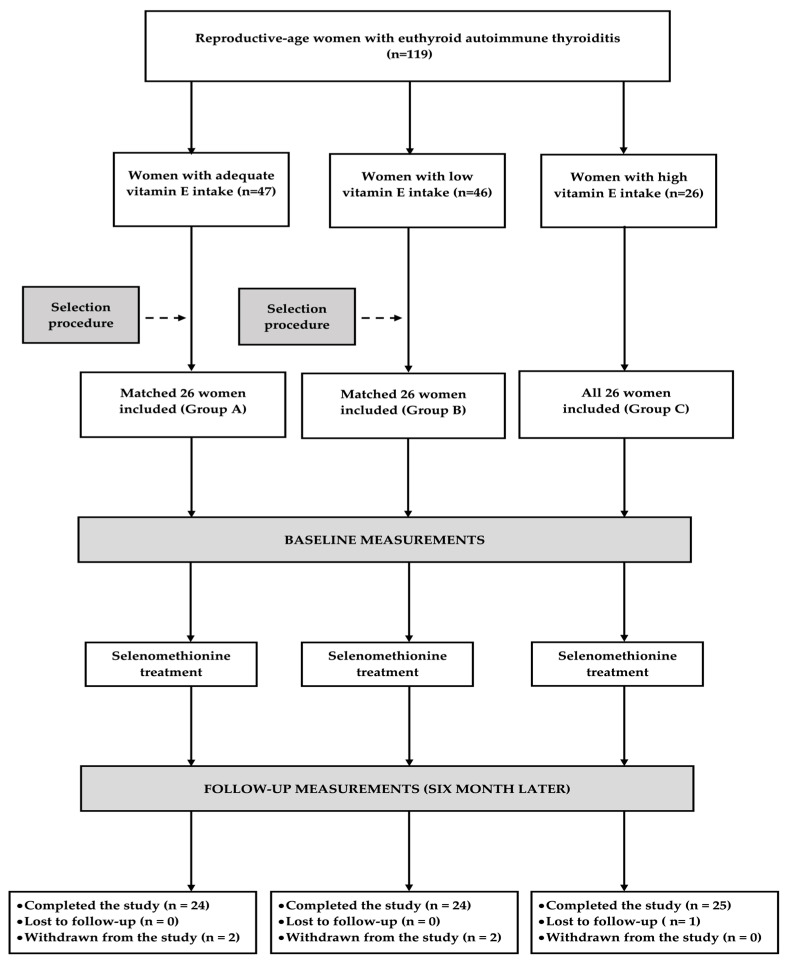
Enrollment and progression of participants through the study.

Participants were assigned to one of three study groups, with adequate (group A), low (group B), and high (group C) vitamin E intake, respectively. An *a priori* sample size analysis showed that the estimated sample size required to detect a given effect size (a 20% change in the total FSFI score, the primary study endpoint), with 80% statistical power and a 5% significance level, was 69 women (22 per group). The effect size and pooled standard deviation (0.5) were based on the results of our previous study assessing the impact of non-hormonal treatment on sexual functioning in young women with euthyroid autoimmune thyroiditis [35]. To account for potential attrition, the planned sample size was increased to 26 participants per group. Group A included women with vitamin E intake equal to or greater than the recommended daily allowance (15 mg of α-tocopherol) [38] but less than 200 IU. Group B comprised women with intake below the estimated average requirement (12 mg of α-tocopherol) [38]. Because participants with daily vitamin E intake below or only slightly exceeding the recommended daily allowance did not use vitamin E supplements, both threshold values were assumed to correspond to 17.9 and 22.4 IU per day, respectively. Group C included women with high vitamin E intake, defined as a daily intake exceeding 400 IU. It was assumed that 1 mg of α-tocopherol is equivalent to 1.49 IU of the natural form or 2.22 IU of the synthetic form. In contrast, the intake criterion for group C was based on the results of a meta-analysis by Miller et al. [39], which demonstrated an increased risk of all-cause mortality associated with daily vitamin E intake exceeding 400 IU. Individuals with daily intake between 17.9 and 22.4 IU and between 200 IU and 400 IU were not eligible for enrollment. All individuals with high vitamin E intake were assigned to group C. Participants in groups A and B were selected from larger eligible cohorts using a computerized matching algorithm to ensure comparability among all groups with respect to age, TPOAb titers, and TSH concentrations (Figure 1).

### 2.2. Study Design

Throughout the six-month study period, all participants received supplemental selenomethionine at a daily dose of 200 μg and were instructed to maintain their usual lifestyle during the study. Adherence to selenomethionine supplementation was monitored every two months through pill counts and participant interviews. The intake of vitamin D, vitamin E, and selenium was assessed based on seven-day dietary recalls. The content of these micronutrients was calculated using information on the type, composition, and amount of consumed foods and dishes, with reference to the recommended tables [40]. This assessment was conducted four times: prior to the start of the study (to determine eligibility for the study group) and every two months (at each follow-up visit) during treatment with selenomethionine. Intake of vitamin D, vitamin E, and selenium over the course of the treatment was calculated as the mean of the obtained results. Total micronutrient intake was determined by combining dietary sources with supplemental intake from tablets or capsules. Concomitant medications—including nonsteroidal anti-inflammatory drugs, paracetamol, hypnotics, antidiarrheal agents, laxatives, antitussives, and antibiotics—were permitted only if taken for ≤14 days and discontinued at least two months prior to the final visit.

### 2.3. Laboratory Assays

All laboratory measurements were performed at baseline and repeated six months later, at the end of the intervention. Venous blood was drawn from the antecubital vein between 8:00 and 9:00 a.m. following a 12 h overnight fast. Samples were collected during the early follicular phase, specifically between cycle days 2 and 5. Before venipuncture, participants rested in a seated position for at least 30 min. Each assay was performed in duplicate, and the mean of the two measurements was used to ensure accuracy. Laboratory personnel were blinded to participants’ group assignments. Serum 25OHD, TSH, free thyroid hormones, estradiol, testosterone, prolactin, and titers of TPOAb and TgAb were measured by direct chemiluminescence using acridinium ester technology (ADVIA Centaur XP, Siemens Healthcare Diagnostics, Munich, Germany). TSH and free thyroid hormone levels were further used to calculate thyroid homeostasis parameters, including Jostel’s TSH index and SPINA indices for thyroid secretory capacity (SPINA-GT) and deiodinase activity (SPINA-GD) [41,42].

### 2.4. Questionnaires

Questionnaire data were collected just after blood sampling, and throughout this period, neither participants nor investigators had access to the results of the biochemical tests. The questionnaires were filled out by participants in a private setting, without guidance or input from the study personnel.

The first questionnaire was aimed at obtaining general information on the participants’ sociodemographic characteristics.

The second questionnaire, FSFI, is a multidimensional self-reported scale for the evaluation of the sexual function of women during the last 4 weeks [43]. It includes 19 questions that examine women’s sexual function in six independent domains: sexual desire (questions 1 and 2), arousal (questions 3, 4, 5, and 6), lubrication (questions 7, 8, 9, and 10), orgasm (questions 11, 12, and 13), sexual satisfaction (questions 14, 15, and 16), and pain (questions 17, 18, and 19). Questions are scored on a 5-point or 6-point Likert scale ranging from 0 to 5 or 1 to 5 within each domain, with the option of “no sexual activity” (0) for four of the domains (arousal, lubrication, orgasm, and satisfaction) [44]. The total score was obtained from the sum of the items in each domain multiplied by the domain factor (0.6 for desire, 0.3 for arousal and lubrication, and 0.4 for orgasm, satisfaction, and pain) [43]. The total FSFI score ranges between 2 and 36, with higher values representing better sexual function. Participants scoring below 26.55 were classified as having sexual dysfunction, while those scoring above this threshold were considered to have normal sexual function [43].

The Beck Depression Inventory-II (BDI-II), the last questionnaire completed by participants, is a 21-item self-administered inventory designed to measure the intensity of depressive symptoms [45]. The items are designed to align with the diagnostic criteria for depressive disorders as defined in the fourth edition of the Diagnostic and Statistical Manual of Mental Disorders [46]. Each item was scored on a 0–3 scale, with 0 indicating absence and 3 indicating severe symptoms. Item scores were then summed to produce a total score ranging from 0 to 63, where higher scores reflect greater severity of depressive symptoms. Scores were interpreted as indicating no depression (0–13), mild depression (14–19), moderate depression (20–28), or severe depression (29 and above) [45].

### 2.5. Statistical Analysis

All continuous variables were logarithmically transformed to correct for non-normality, stabilize variance, and reduce the influence of extreme values (outliers). Between-group comparisons at the same time point, as well as comparisons of percentage changes from baseline, were performed using one-way analysis of variance followed by Bonferroni’s post hoc multiple comparison test. Pre- and post-treatment values within the same group were compared using Student’s paired t-test. Categorical data were analyzed using the chi-square test. The strength of associations between variables was assessed using Pearson’s correlation coefficient for two continuous variables, the phi coefficient for one continuous and one categorical variable, and the point-biserial correlation for two categorical variables. To determine whether the measured variables are associated with sexual function, multivariate linear regression analysis adjusted for age, smoking status, body mass index, and blood pressure was performed using total FSFI scores and FSFI domain scores as dependent variables, and micronutrient intake, antibody titers, hormone levels, and calculated parameters of thyroid homeostasis as independent variables. In addition, a Sobel test was conducted to determine whether the assessed hormones had significant mediating effects on the relationship between metformin-induced changes in thyroid antibody titers and sexual function. Two-tailed *p*-values after correction for multiply testing less than 0.05 were considered statistically significant.

## 3. Results

### 3.1. Baseline Demographic, Anthropometric, and Laboratory Characteristics of Study Groups

At the initiation of the study, the compared patient groups differed with respect to daily vitamin E intake and serum testosterone concentrations. No differences were observed between the study groups in terms of age, smoking status, physical activity, educational level, occupational activity, number of sexual partners, number and duration of marriages, number of deliveries and miscarriages, body mass index, or blood pressure. In addition, there were no differences in mean daily vitamin D or selenium intake; titers of thyroid antibodies; serum concentrations of TSH, free thyroxine, free triiodothyronine, estradiol, prolactin, or 25OHD; or in the values of Jostel’s index, SPINA-GT, SPINA-GD, total FSFI score, and BDI-II score (Table 1).

### 3.2. Study Participant Progression

Seventy-three women completed the study, representing 94% of the enrolled participants. A post hoc evaluation indicated that the study achieved adequate statistical power (0.86). The most common reason for discontinuation—observed in three patients (one in group A and two in group B)—was nonadherence to dietary recommendations. One patient from group A withdrew due to pregnancy; the course of the pregnancy was uncomplicated and resulted in the birth of a healthy child. In addition, one patient from group C discontinued follow-up visits without providing a reason (Figure 1). Selenomethionine treatment was well tolerated. Adverse events were reported in two patients (increased irritability and more frequent urination, respectively); these symptoms were mild, occurred only during the first week of treatment, and subsequently resolved. Mean daily dietary vitamin D and selenium intake with food (not including selenium contained in the selenometionine preparation) was 18 ± 10 µg and 42 ± 12 µg in group A, 18 ± 9 µg and 40 ± 16 µg in group B, and 21 ± 12 µg and 46 ± 17 µg in group C, respectively, and did not differ from baseline values. Mean daily dietary intake of vitamin E during the study differed among the groups (*p* < 0.001), amounting to 36 ± 50 mg, 8 ± 2 mg, and 950 ± 261 mg, respectively. However, within each group, vitamin E intake did not differ from the daily intake at baseline. At the end of the study, 25OHD levels (118.2 ± 17.4 nmol/L in group A, 108.2 ± 20.1 nmol/L in group B, and 111.0 ± 19.4 nmol/L in group C) were unchanged from baseline and did not differ between groups.

### 3.3. Biochemical Variables

**Figure 2:**
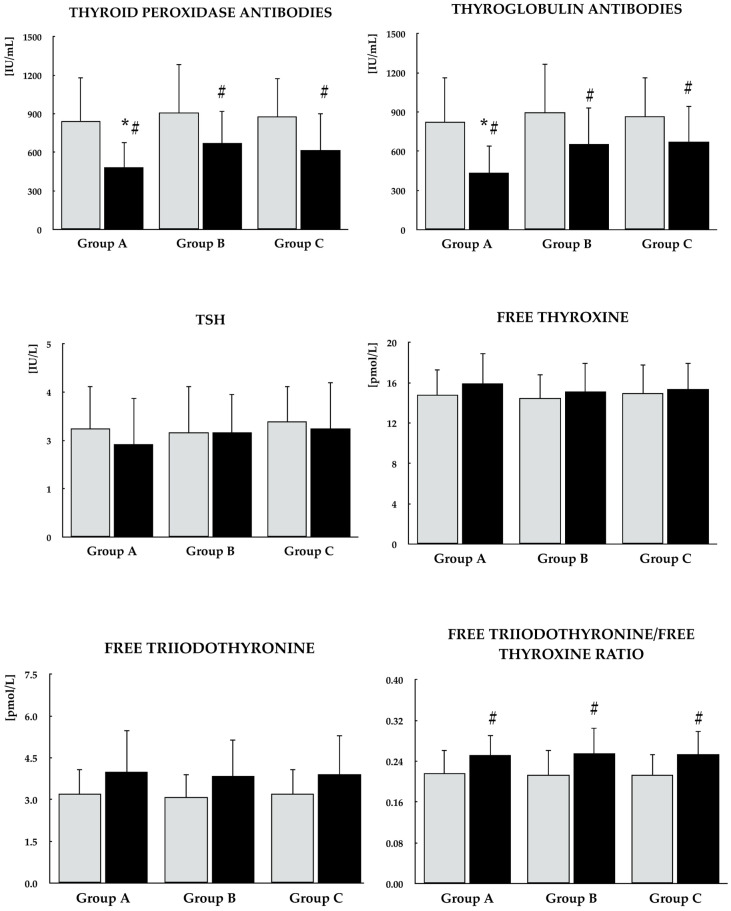
Effect of selenomethionine on thyroid antibody titers and hypothalamic–pituitary–thyroid axis activity in reproductive-age women with euthyroid autoimmune thyroiditis, stratified by vitamin E status. Group A: adequate vitamin E intake; Group B: low vitamin E intake; Group C: high vitamin E intake. Values are expressed as mean and standard deviation. Free triiodothyronine/free thyroxine ratio is a dimensionless parameter. Gray bars indicate values at study entry, while black bars indicate values at study end. * *p* < 0.05 vs. the corresponding values in groups B and C. ^#^ *p* < 0.05 vs. baseline values within the same group.

**Figure 3:**
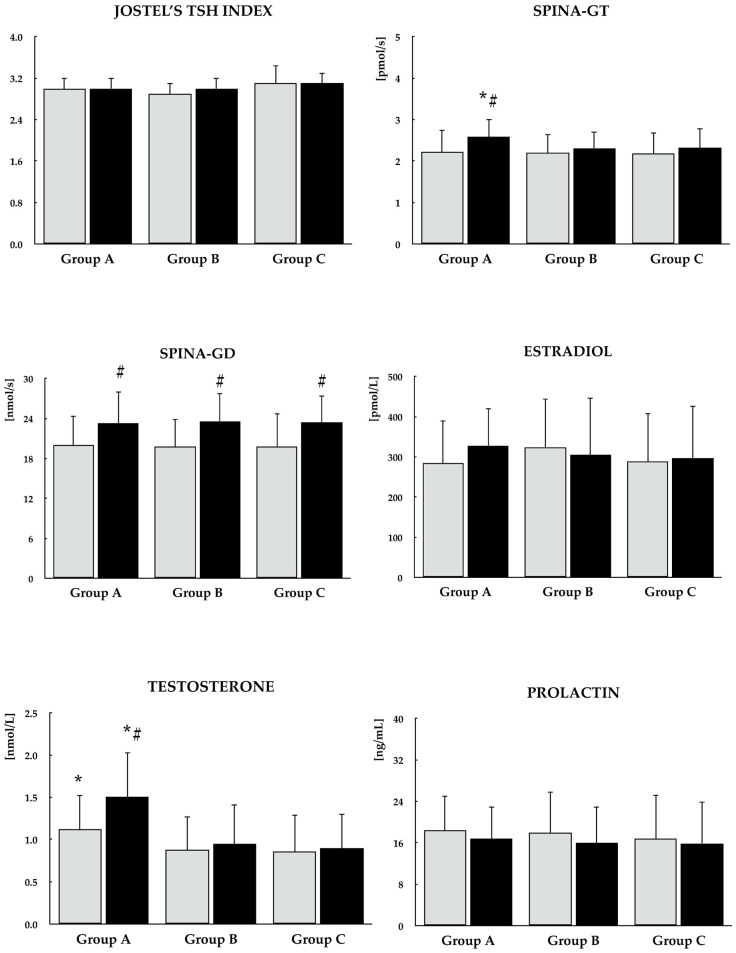
Effect of selenomethionine on calculated parameters of thyroid homeostastis and the remaining hormones in reproductive-age women with euthyroid autoimmune thyroiditis, stratified by vitamin E status. Group A: adequate vitamin E intake; Group B: low vitamin E intake; Group C: high vitamin E intake. Values are expressed as mean and standard deviation. Jostel’s TSH index is a dimensionless parameter. Gray bars indicate values at study entry, while black bars indicate values at study end. * *p* < 0.05 vs. the corresponding values in groups B and C. ^#^* p* < 0.05 vs. baseline values within the same group.

**Figure 4:**
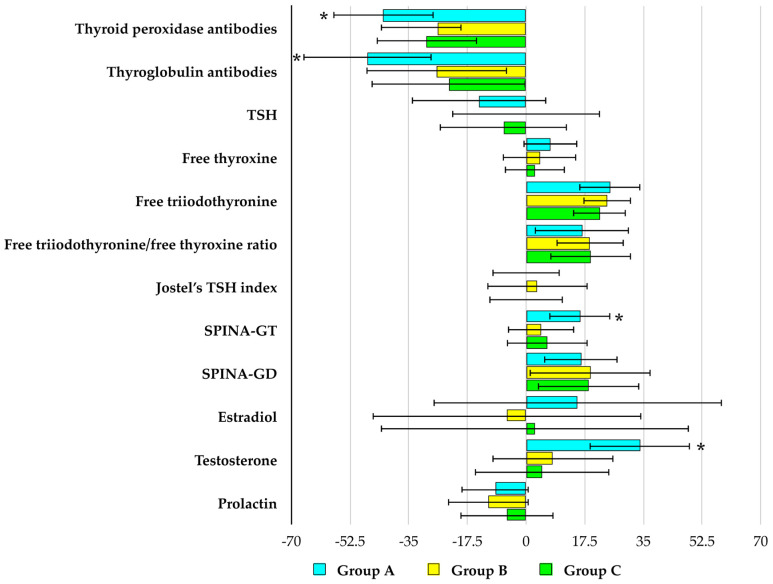
Percentage changes from baseline in biochemical variables in reproductive-age women with euthyroid autoimmune thyroiditis, stratified by vitamin E status. Values are expressed as mean ± standard deviation. Group A: adequate vitamin E intake; Group B: low vitamin E intake; Group C: high vitamin E intake. * *p* < 0.05 vs. the corresponding values in groups B and C.

In all studied groups, selenomethionine caused a reduction in TPOAb and TgAb titers, as well as an increase in the ratio of free triiodothyronine to free thyroxine and in the SPINA-GD index. An increase in the SPINA-GT index and testosterone concentration was observed exclusively in group A. Differences were noted between group A and the other groups in the percentage changes in both antibody titers, testosterone levels, as well as in the SPINA-GT index during treatment. By the end of the study, the values of all these parameters differed between group A and the remaining groups. No changes were observed in any of the studied groups with respect to the other parameters (TSH, free thyroxine, free triiodothyronine, Jostel’s TSH index, estradiol, and prolactin) (Figure 2, Figure 3 and Figure 4).

### 3.4. Sexual Functioning

**Figure 5:**
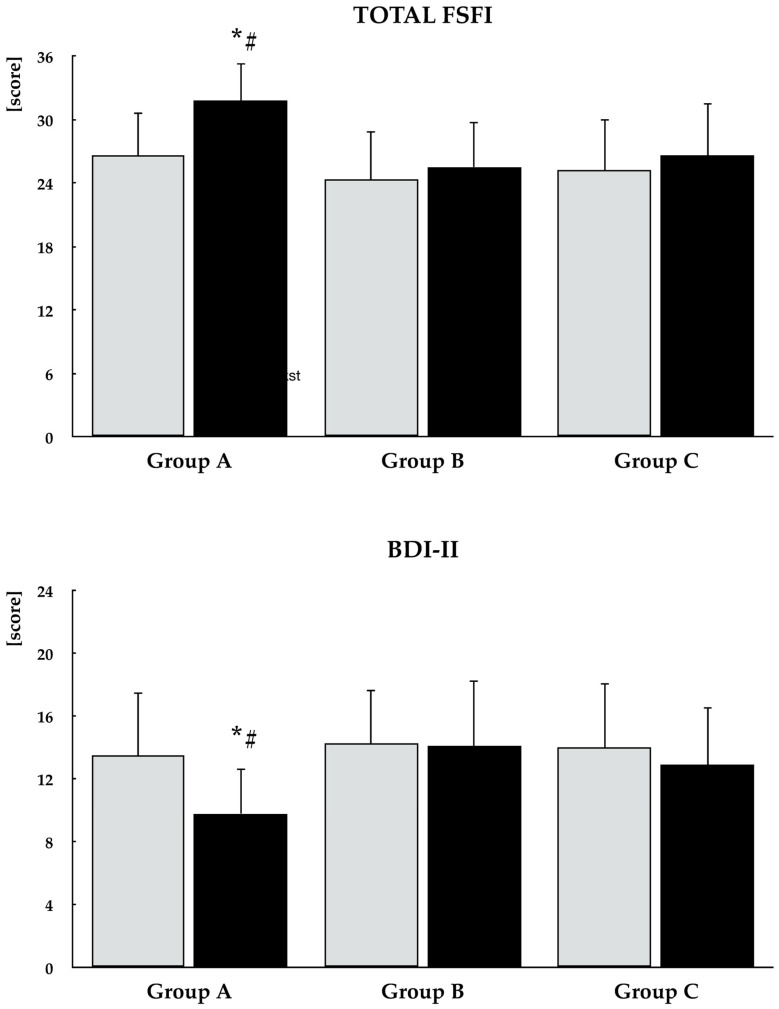
Effect of selenomethionine on total FSFI and BDI-II scores in reproductive-age women with euthyroid autoimmune thyroiditis, stratified by vitamin E status. Group A: adequate vitamin E intake; Group B: low vitamin E intake; Group C: high vitamin E intake. Values are expressed as mean and standard deviation. Gray bars indicate values at study entry, while black bars indicate values at study end. * *p* < 0.05 vs. the corresponding values in groups B and C. ^#^ *p* < 0.05 vs. baseline values within the same group.

**Figure 6:**
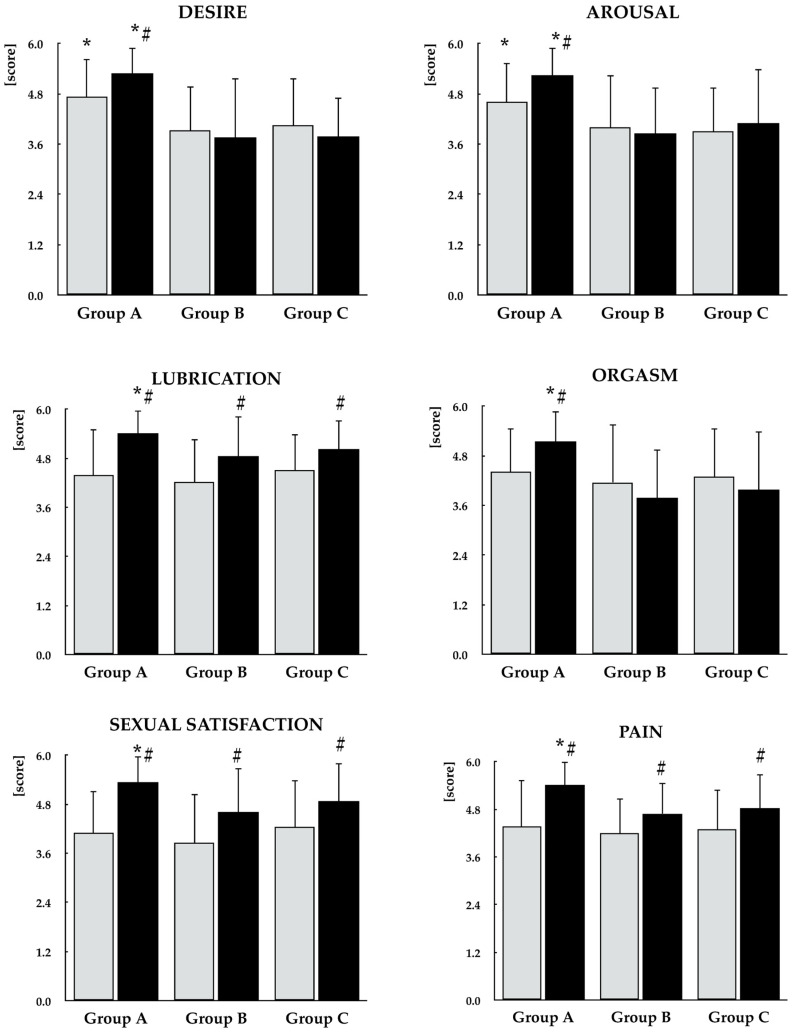
Effect of selenomethionine on components of sexual function in reproductive-age women with euthyroid autoimmune thyroiditis, stratified by vitamin E status. Group A: adequate vitamin E intake; Group B: low vitamin E intake; Group C: high vitamin E intake. Values are expressed as mean and standard deviation. Gray bars indicate values at study entry, while black bars indicate values at study end. * *p* < 0.05 vs. the corresponding values in groups B and C. ^#^ *p* < 0.05 vs. baseline values within the same group.

**Figure 7:**
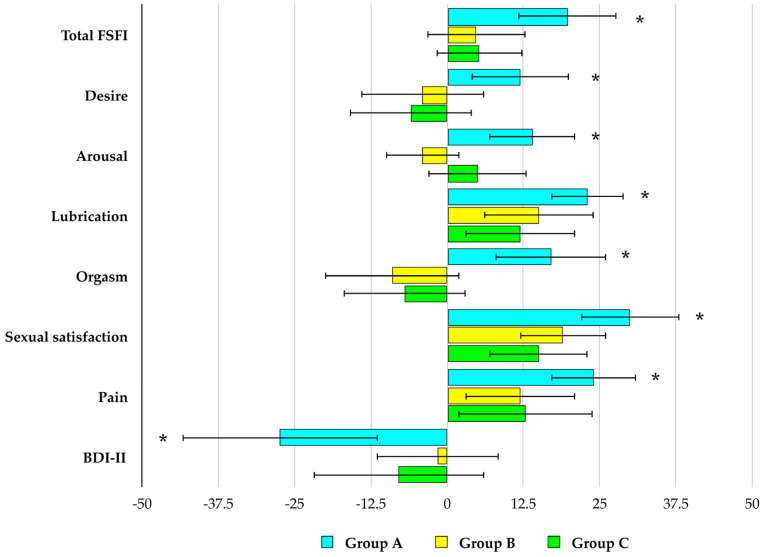
Percentage changes from baseline in sexual functioning and depressive symptoms in reproductive-age women with euthyroid autoimmune thyroiditis, stratified by vitamin E status. Values are expressed as mean ± standard deviation. Group A: adequate vitamin E intake; Group B: low vitamin E intake; Group C: high vitamin E intake. * *p* < 0.05 vs. the corresponding values in groups B and C.

At baseline, group A differed from the other study groups with respect to the desire and arousal domain scores. In group A, selenomethionine treatment resulted in an increase in the total FSFI score as well as in all individual domain scores. In groups B and C, the intervention led to improvements in the lubrication, sexual satisfaction, and pain domains, with no significant effects observed in the remaining FSFI domains. Percentage increases in the total FSFI score and all domain scores were greatest in group A and did not differ significantly between the other study groups. At the end of the study, differences between group A and the other groups were observed in the total FSFI score and all domain scores. A reduction in the proportion of women with a total FSFI score ≤ 26.55 was observed only in group A (Figure 5, Figure 6 and Figure 7, Table 2).

### 3.5. Depressive Symptoms

At baseline, there were no statistically significant between-group differences in the total BDI-II score or in the proportion of patients presenting with depressive symptoms. Selenomethionine treatment led to a reduction in both the total BDI-II score and the proportion of affected patients only in group A. At the end of the study, group A differed significantly from the other groups with respect to the total BDI-II score and the proportion of patients with overall and mild depressive symptoms (Figure 5 and Figure 7, Table 2).

### 3.6. Correlations, Multivariate Regression Analysis, and Mediation Analysis

At baseline, positive correlations were identified between the total FSFI score and all domain scores, as well as baseline daily dietary intake of vitamin E (r = 0.27 [*p* < 0.05] to 0.39 [*p* < 0.01]) and selenium (r = 0.24 [*p* < 0.05] to 0.38 [*p* < 0.01]). The effect of selenomethionine on TPOAb and TgAb titers correlated with their baseline titers (r = 0.51 [*p* < 0.001] to 0.65 [*p* < 0.001]), changes in SPINA-GT (r = 0.26 [*p* < 0.05] to 0.42 [*p* < 0.01]) and changes in testosterone levels (r = 0.29 [*p* < 0.05] to 0.40 [*p* < 0.01]). Increases in the total FSFI score and all domain scores were positively correlated with changes in thyroid antibody titers. In addition, positive correlations were observed between increases in the desire and arousal domain scores and increases in testosterone levels, as well as between increases in the lubrication domain score and baseline estradiol levels (Table 3). In groups A and B, the total FSFI score and all domain scores were positively correlated with daily vitamin E intake (Table 4). Finally, the effect of selenomethionine on the total BDI-II score was positively correlated with changes in the total FSFI score and all domain scores (Table 5).

In linear regression analysis, selenomethionine-induced changes in TPOAb titers were independently associated with improvements in total and domain sexual functioning. A similar predictive role was observed for mean daily vitamin E intake during the study, but only in analyses restricted to groups A and B (Table 6).

In the Sobel test, only testosterone was identified as a significant mediator of the associations between selenomethionine-induced changes in TPOAb titers and sexual desire (z-scores ranging from 2.41 [*p* < 0.01] to 2.98 [*p* < 0.001]) and arousal (z-scores ranging from 2.15 [*p* < 0.05] to 2.76 [*p* < 0.01]). No other hormones demonstrated significant mediating effects on any domain of sexual functioning.

## 4. Discussion

In line with previous meta-analyses demonstrating the efficacy of selenium compounds [18,19,20], selenomethionine lowered thyroid antibody titers across all patient groups. Notably, and for the first time, this effect was most pronounced in the group receiving the recommended intake of vitamin E. This was also the only group in which an increase in SPINA-GT was observed, despite no changes in hypothalamic–pituitary–thyroid axis hormone levels. SPINA-GT is a marker of the thyroid’s maximal capacity to secrete endogenous thyroxine and is considered more sensitive than measurements of TSH or free thyroid hormone fractions [41,42]. Our findings suggest that the increase in SPINA-GT reflects the strongest effect of selenomethionine on the autoimmune process in the thyroid, resulting in the greatest reduction in inflammatory infiltration and normalization of thyrocyte function in the group with adequate vitamin E intake. The lack of a significant effect on circulating thyroid hormone levels may be related to the normal activity of the hypothalamic–pituitary–thyroid axis, indicating only a relatively minor thyroid functional impairment at baseline. If this interpretation is correct, the benefits of selenomethionine supplementation, in combination with adequate vitamin E intake, may be even greater in individuals who develop hypothyroidism—particularly if treatment is initiated before the onset of advanced fibrotic changes in the thyroid.

The present study is the first to demonstrate that the effect of selenomethionine on sexual function in women with autoimmune thyroiditis is influenced by the intake of a natural antioxidant. Improvements in the global FSFI score, as well as across all domains of the questionnaire, were observed exclusively in the group receiving recommended vitamin E supplementation. In cases of both insufficient and excessive vitamin E intake, improvements were markedly less pronounced and were limited to lubrication, sexual satisfaction, and pain. These findings suggest that the interaction between selenomethionine and vitamin E in terms of sexual function—and mood—follows an inverted U-shaped relationship. Furthermore, except in cases of excessive vitamin E intake, improvements in sexual functioning were positively correlated with daily vitamin E consumption. These results allow several conclusions. First, the beneficial effect of selenomethionine on sexual functioning in reproductive-age women with autoimmune thyroiditis is observed even when hypothalamic–pituitary–thyroid axis activity remains within the normal range. Second, both vitamin E deficiency and excessive intake impair the effects of selenomethionine and should be avoided in women with sexual dysfunction. Third, abnormal vitamin E intake may, at least in part, explain the previously observed weaker effect of selenomethionine on female sexual responsiveness compared with exogenous vitamin D [35]. Finally, the absence of significant improvement in sexual functioning among women receiving selenomethionine underscores the importance of assessing vitamin E status and implementing appropriate dietary modifications when abnormalities are detected.

The study results suggest a beneficial effect of adequate vitamin E intake on the efficacy of selenomethionine, both in terms of the severity of the autoimmune process and the assessed clinical outcomes. In linear regression analysis, mean daily vitamin E intake was independently associated with improvements in sexual functioning induced by selenomethionine, except at high intake. Moreover, correlations were observed between daily vitamin E intake and the effects of selenomethionine on all aspects of sexual function in the low- and adequate-intake groups. In the former group, this finding is a logical consequence of the reduced efficacy of the compound compared with the adequate-intake group, as well as the application of an arbitrary threshold distinguishing adequate from low intake. These results indicate that even within this subgroup, differences in treatment effect magnitude are present and that even a modest increase in vitamin E intake may enhance the effects of selenomethionine. An even more interesting observation was the presence of similar correlations in women with adequate vitamin E intake. This may suggest that the effect of selenomethionine is further enhanced by supplementation with moderate doses of vitamin E, even when dietary intake already exceeds the recommended dietary allowance. This finding supports the rationale for combined therapy with selenomethionine and exogenous vitamin E. Verification of this hypothesis will be a focus of future research conducted by our team. The absence of a correlation in the high-intake group, however, suggests that vitamin E intake exceeding 400 IU per day should be avoided, although it does not provide evidence that the potentially adverse effects of very high vitamin E intake are dose-dependent.

The findings of the present study support the involvement of two principal mechanisms responsible for the improvement in sexual functioning observed in the study population, as well as for the differences in the effects of selenomethionine between the individual groups. The first mechanism is the direct effect of this compound on the severity of thyroid autoimmunity. Evidence for this role is provided by the presence of correlations between changes in thyroid antibody titers during treatment and improvements in sexual functioning. It should be emphasized that no analogous correlations were observed between sexual functioning and markers of the hypothalamic–pituitary–thyroid axis, including those that changed during selenomethionine treatment (SPINA-GT, the free triiodothyronine/free thyroxine ratio, and SPINA-GD). Moreover, multivariate regression analysis showed that TPOAb titers were an independent and significant predictor of sexual functioning in selenomethionine-treated women. These findings indicate that the effect of reducing the severity of thyroid autoimmunity on female sexual response cannot be explained by changes in peripheral conversion of thyroxine to triiodothyronine, nor—within the group receiving adequate vitamin E supplementation—by alterations in thyroid hormone secretion. Additional support for this interpretation is provided by the observation that correlations between reductions in thyroid antibody titers and improvements in lubrication, sexual satisfaction, and pain were stronger than those observed for other domains of sexual functioning. This may explain why, in the groups with insufficient and excessive vitamin E intake, improvement was limited exclusively to these three domains. Conversely, the relatively modest reduction in antibody titers in both these groups may have been insufficient to induce improvements in libido, arousal, and orgasm. However, because the effect on TPOAb and TgAb titers correlated with their baseline values, it cannot be excluded that such effects may be observed in women with autoimmune thyroiditis in the hypothyroid stage, in whom thyroid antibody titers are higher than in euthyroid disease [2].

The second mechanism that may account for the observed changes involves testosterone. Higher baseline testosterone concentrations in the group receiving adequate vitamin E supplementation, as well as a further increase during treatment observed exclusively in this group, adequately explain the baseline differences in desire and arousal between the groups (despite similar baseline thyroid antibody titers), as well as the subsequent improvement in both domains among women with adequate vitamin E supplementation. In line with this, mediation analysis showed that testosterone may mediate the effects of selenomethionine on libido and arousal, but not on other aspects of female sexual function. Further confirmation of this explanation is provided by the presence of positive correlations between changes in testosterone concentrations and improvements in both domains, as well as by the fact that these correlations were stronger than the corresponding correlations with changes in thyroid antibody titers. Moreover, testosterone is regarded as the most important hormone for normal female sexual functioning, exerting its most pronounced effects on libido and excitement [47,48]. Among the hormones not associated with the hypothalamic–pituitary–thyroid axis, testosterone was the only one whose concentration differed between the groups both before and after completion of the study. This observation is consistent with our previous findings indicating higher testosterone levels in women with autoimmune thyroiditis receiving selenomethionine [35]. However, this fact cannot account for the baseline differences in testosterone concentrations, as daily dietary selenium intake was comparable across all groups. Therefore, an association with differences in vitamin E intake appears more likely. Nevertheless, the present findings are inconsistent with the results of a meta-analysis by Yalle-Vásquez et al. [49], who reported a reduction in testosterone levels in women with polycystic ovary syndrome. These discrepancies may be attributable to differences in baseline testosterone concentrations, as hyperandrogenism is a characteristic feature of polycystic ovary syndrome [50]. It is possible that low testosterone levels, particularly in the group with insufficient vitamin E intake, result from the adverse effects of oxidative stress on the hormonal activity of theca cells and, possibly, the adrenal zona reticularis—effects that would not be adequately counterbalanced by the action of vitamin E [51].

Despite the established association of prolactin with the induction and progression of autoimmune thyroiditis and the role of hyperprolactinemia in sexual dysfunction among women of reproductive age [52,53,54], prolactin concentrations in all study groups remained within the normal range, did not change during the study, and were not correlated with sexual functioning. For analogous reasons—namely, comparable baseline levels and the absence of treatment-related changes—the observed findings cannot be explained by effects of estradiol or 25OHD. Although a weak positive correlation between estradiol levels and lubrication scores was observed, this finding most likely reflects the physiological role of estrogens in regulating normal vaginal lubrication [55]. Finally, the lack of association with 25OHD, despite previously reported effects of vitamin D supplementation on sexual functioning in women with autoimmune thyroiditis, is most likely attributable to the inclusion criteria applied in the present study, which restricted participation to women with normal vitamin D homeostasis [35]. The regression analysis also did not provide evidence of an association between these biochemicals and sexual functioning.

As the present study was confined to the evaluation of thyroid autoimmunity markers, thyroid function parameters, and the concentrations of other hormones, the findings do not permit confirmation or exclusion of other mechanisms that may contribute to the regulation of female sexual function, although these merit brief discussion. Normal female sexual response depends on adequate vascularization and innervation of the genital tissues [56]. Both vitamin E and selenium compounds, largely because of their antioxidant properties, play important roles in regulating vascular blood flow and maintaining central and peripheral nervous system function, thereby protecting against atherosclerotic changes and neurotoxic effects associated with insufficient intake [57,58,59,60]. Notably, these mechanisms could also account for the diminished efficacy of selenomethionine observed in the context of excessive vitamin E supplementation. It should be emphasized that excessive intake of vitamin E appears to be associated with an adverse effect on the risk of progression of ischemic heart disease and the occurrence of stroke (particularly hemorrhagic) [61,62], while a detrimental effect on overall mortality has been observed when daily intake exceeds 400 IU [39], which in our study served as the criterion for inclusion in the high vitamin E intake group. However, because genital perfusion and neural innervation were not directly assessed in this study, the contribution of these pathways cannot be determined and warrants further investigation, which will be the focus of future studies.

Another important finding of this study was the high prevalence of patients meeting diagnostic criteria for depression based on BDI-II scores (53%); however, depressive symptoms were mild in most cases. These results corroborate previous findings from both other research centers and our own group, indicating that women with autoimmune thyroiditis are predisposed to depressed mood even when hypothalamic–pituitary–thyroid axis function remains normal [5,12,63]. In contrast to earlier studies, however, our findings exclude an association between these observations and disturbed vitamin D homeostasis, which is a frequent abnormality coexisting with Hashimoto’s disease and which itself predisposes to the development of depressive symptoms [64,65]. A particularly noteworthy finding was the demonstration of a beneficial effect of selenomethionine on mood. This effect, reflected by both a reduction in BDI-II scores and a lower proportion of patients meeting criteria for mild depression, was observed exclusively among women receiving adequate vitamin E supplementation. The antidepressant effect of selenium observed in our study is consistent with the findings of a recently published meta-analysis; however, that analysis predominantly included studies involving participants without thyroid disease [66]. Notably, although vitamin E also demonstrated antidepressant properties, its benefits were primarily reported in studies using doses comparable to those applied in the present investigation [64,67]. The comparable magnitude of selenomethionine’s effects on mood and sexual function, together with the observed correlations between changes in BDI-II scores and improvements in overall sexual functioning and its individual domains, suggests a shared underlying mechanism. These findings point to the role of interactions between selenomethionine and vitamin E homeostasis in mediating both effects. Nevertheless, the observed correlations do not permit determination of causality, as improvement in mood may be either a consequence or a determinant of improved sexual functioning. The present data provide at least partial support for the former interpretation. Changes in BDI-II scores were correlated with sexual functioning, whereas—unlike sexual outcomes—they showed no association with the thyroid-level anti-inflammatory or hormonal effects of selenomethionine therapy. Given that correlations between the effects of selenomethionine on mood and sexual functioning were only moderate, it is reasonable to assume that mood improvement depends on additional contributing factors and cannot be explained by the epidemiologic variables evaluated in this study. A synergistic antioxidant and anti-inflammatory effect of selenium and vitamin E therefore appears to be a more plausible mechanism [26,68]. This interpretation is further supported by evidence that both oxidative stress and chronic inflammation contribute to the pathogenesis of autoimmune thyroiditis as well as depression [21,69,70,71,72].

The main limitation of the study is the absence of measurements of serum selenium and vitamin E levels, as well as the lack of assessment of oxidative stress-related indicators (including malondialdehyde, glutathione geroxidase, and thioredoxin reductase). Without an objective assessment of these parameters, it is difficult to determine the participants’ actual micronutrient status and oxidative stress, and the interpretation of the findings remains largely speculative. Indirect evidence suggests, however, that the participants were selenium-deficient prior to the initiation of the study and that this deficiency was no longer present following selenomethionine supplementation. The study was conducted in a region with documented selenium deficiency [36], and throughout the study period the population had low dietary selenium intake. Measurements of circulating selenium concentrations performed in 20 study participants during the six months preceding study initiation (conducted in different laboratories) demonstrated that 16 of them (80%) had values below the reference range for the respective laboratory. Lastly, all evaluated groups exhibited low and comparable baseline values of the free triiodothyronine/free thyroxine ratio and the SPINA-GD index. Both parameters reflect the global activity of peripheral deiodinases, which are chemically classified as selenoproteins [42,73], and their low baseline values suggest reduced deiodinase activity in the participants, which appears to be associated with selenium deficiency. In line with this, selenomethionine supplementation was followed by an increase in the free triiodothyronine/free thyroxine ratio and the SPINA-GD index. Notably, the percentage changes from baseline did not differ significantly between the groups, which may indicate that the interaction between selenium and vitamin E is primarily pharmacodynamic in nature, rather than related to the effect of vitamin E on selenium pharmacokinetics. While the lack of selenium concentration assessment imposes interpretative constraints on our study, there are two important considerations in evaluating selenium status and other selenium biomarkers in the context of high-dose selenomethionine supplementation. First, due to the nonspecific incorporation of selenomethionine into various proteins, the increase in selenium concentration in serum (or whole blood) is greater than that observed with comparable doses of inorganic selenium compounds [74]. Second, as certain enzymes become saturated, the relationship between selenomethionine intake and selenium or selenoprotein levels is nonlinear [75]. Therefore, it cannot be excluded that the observed associations between the effects on the assessed biochemical markers, sexual function, and mood and vitamin E intake may differ from those observed with inorganic selenium compounds (selenite and selenate). To address the lack of serum vitamin E measurements, intake was assessed prior to study initiation and subsequently on three occasions; the total duration of intake monitoring constituted one eighth of the participants’ study participation period. In addition, participants with borderline intake levels (17.9–22.4 IU and 200–400 IU), as well as those with comorbidities or receiving other medications that could potentially affect the pharmacokinetics of this vitamin, were excluded. Although these measures increase the likelihood of obtaining three cohorts differing in vitamin E status, future studies evaluating interactions between selenium compounds and vitamin E should incorporate more precise parameters to characterize vitamin E homeostasis. It should also be noted that, due to its storage in adipose tissue, assessment of serum vitamin E levels—while recommended—does not always accurately reflect total body stores or its distribution across peripheral tissues, thereby justifying the need to explore additional parameters of vitamin E metabolism [76,77].

The design of the study does not permit identification of the processes responsible for the interaction between selenomethionine and vitamin E. As indicated earlier, vitamin E does not appear to influence the uptake, distribution, biotransformation, or clearance of this compound. The interpretation of the findings is further hindered by the absence of a dedicated receptor for this vitamin, as well as by the presence of multiple structurally distinct forms of vitamin E [25]. Most likely, this interaction occurs at the level of oxidation–reduction (redox) reactions. Vitamin E acts as a direct lipid-phase free radical scavenger [23], whereas selenium functions as an essential component of antioxidant enzymes (such as glutathione peroxidase and thioredoxin reductase) [68]. Together, these compounds provide complementary and synergistic antioxidant protection in the thyroid gland [21,22], and possibly also in structures involved in the regulation of sexual function and emotions. This may explain the stronger effect of selenomethionine in the context of adequate vitamin E intake, compared with conditions of insufficient intake, which may create an environment that is not conducive to the manifestation of synergy between the two antioxidants. It is also important to emphasize that redox-level interactions may further explain the weak effect of selenomethionine in women with excessive vitamin E intake. In such cases, an antioxidant-to-prooxidant transition may occur as a result of the accumulation of the tocopheroxyl radical, which, under favorable conditions (e.g., metabolic dysregulation, the presence of transition metals, or specific genetic backgrounds), may exhibit pro-oxidant activity, thereby opposing the effects of selenium compounds [78]. However, the proposed mechanism is based on in vitro studies, has not been verified in the present study, and is therefore unclear in its applicability to women with autoimmune thyroiditis. Consequently, these results require confirmation in studies incorporating markers of redox reactions.

The results of the correlation and regression analyses suggest a statistically significant interaction between selenomethionine and vitamin E in relation to sexual response among young women. The attenuation of selenomethionine’s effects on mood under conditions of both low and high vitamin E intake was likely partly secondary to a less pronounced improvement in sexual functioning, as indicated by the pattern of the observed correlations. Interestingly, despite clear differences in the strength of the compound’s effects on the severity of the autoimmune process and on thyroid secretory function, no correlation was observed between vitamin E intake and the degree of reduction in anti-thyroid antibody titers or changes in thyroid secretory capacity. This may be attributable to the fact that global sexual functioning, rather than laboratory parameters, constituted the primary endpoint of the study and was used to determine the sample sizes of the respective patient groups. It may also reflect the selection process employed, which aimed to achieve a comparable level of thyroid autoimmunity at baseline. Finally, extreme values—frequently observed in measurements of anti-thyroid antibody titers in individuals with Hashimoto’s disease [2]—as well as other confounding variables, may have influenced the correlation analyses, thereby masking existing associations.

It is evident that the inclusion of placebo or no-intervention control groups would have strengthened the interpretability of the results. However, this would have required the creation of three additional groups stratified by vitamin E intake (analogous to those receiving selenomethionine), thereby introducing substantial methodological and interpretative challenges. First, such a design would have required the recruitment of a significantly larger number of participants, particularly given that the study groups were matched for age, TPOAb titers, and TSH levels. The power analysis determined the minimum number of participants required per group, and the total enrollment accounted for potential withdrawals during the study. The formation of such groups would have been especially challenging in patients with high vitamin E intake (in the present study, all such patients were included unless they met exclusion criteria). Moreover, the creation of additional groups would increase the risk of error due to multiple comparisons, which rises with the number of groups analyzed. Consequently, a decision was made to employ three cohorts despite the inherent limitations of this approach. Unfortunately, the absence of a placebo or blank control group does not allow us to completely exclude the possibility that the results were influenced by the natural history of thyroiditis or by patient expectations.

Other limitations of the research protocol also warrant consideration. Although the sample size exceeded the minimum required, the small number of participants increases the risk of random error, makes the analysis more sensitive to outliers, may lead to failure to detect potential intergroup differences, and limits the generalizability of the results. Cohort studies are susceptible to bias and confounding, potentially compromising the validity and reliability of the findings [79]. While the FSFI and BDI-II instruments are rigorously validated, their effectiveness is inherently restricted by reliance on subjective assessments. Given that the Polish population has adequate iodine intake due to mandatory iodine supplementation, the results observed here may differ in populations with iodine deficiency [80]. Lastly, although design-stage precautions were taken, the findings may still be affected by regression dilution, arising from gradual physiological changes in the measured outcomes, and/or regression toward the mean, whereby initially extreme values naturally converge toward the overall average over time [81,82].

## 5. Conclusions

Low and excessive vitamin E intake in women with Hashimoto’s thyroiditis is associated with reduced libido and arousal. Although selenomethionine reduces thyroid autoimmunity independently of vitamin E intake, this effect is strongest in individuals consuming the recommended amount. Adequate vitamin E intake is necessary to observe improvements in total FSFI scores and all individual domain scores. In addition to reducing thyroid antibody titers, this effect is partially mediated by increased testosterone levels. Adequate vitamin E intake is also required to manifest the beneficial effects of selenomethionine on mood, which appear to be at least partly secondary to improvements in sexual function. These findings demonstrate, for the first time, that dietary intake of a natural antioxidant may modulate the effects of exogenous selenomethionine on sexual function and depressive symptoms in reproductive-age women with euthyroid autoimmune thyroiditis. They also indicate that both insufficient and excessive vitamin E intake should be avoided. Therefore, optimizing oxidative status appears to be a reasonable therapeutic goal in young women with Hashimoto’s disease who maintain normal hypothalamic–pituitary–thyroid axis function. The pilot design and associated limitations of this study, together with the novelty of the findings, underscore the need for validation in large-scale, methodologically rigorous multicenter studies. Moreover, the results emphasize the importance of exploring the underlying biological mechanisms that may mediate the interaction between selenomethionine and vitamin E.

## Figures and Tables

**Table 1:** Baseline characteristics of study groups: demographic, anthropometric, and laboratory data (intention-to-treat analysis).

Variable	Group A	Group B	Group C
Number of patients	26	26	26
Age (years)	35 ± 7	36 ± 8	34 ± 7
Smokers (%)/Number of cigarettes a day (n)/Smoking duration (months)	38/9 ± 4/95 ± 68	35/7 ± 5/105 ± 70	35/8 ± 5/90 ± 61
Physical activity: total/several times a week/once a week/once a month (%)	81/50/23/8	81/46/23/12	77/46/19/12
Primary or vocational/secondary/university education (%)	8/42/50	12/38/50	12/34/54
Employed/Blue-collar/white-collar/pink-collar workers (%)	92/15/31/46	92/19/35/38	96/19/38/38
Number of sexual partners (n)	2.5 ± 0.9	2.6 ± 1.0	2.3 ± 1.2
Number of marriages (n)/duration of marriages (months)	1.3 ± 0.5/92 ± 40	1.2 ± 0.5/96 ± 36	1.3 ± 0.6/89 ± 46
Number of deliveries (n)/Number of miscarriages (n)	1.3 ± 0.6/0.5 ± 0.3	1.2 ± 0.6/0.5 ± 0.2	1.2 ± 0.5/0.6 ± 0.4
Body mass index (kg/m^2^)	23.7 ± 4.9	24.4 ± 5.0	24.2 ± 4.7
Systolic blood pressure (mm Hg)	123 ± 22	127 ± 18	119 ± 23
Diastolic blood pressure (mm Hg)	76 ± 6	77 ± 6	74 ± 7
Mean dietary vitamin D intake (µg/day)	18 ± 8	17 ± 8	20 ± 10
Mean dietary vitamin E intake (mg/day)	37 ± 40 ^#^	7 ± 3	975 ± 282 *^#^
Mean dietary selenium intake (µg/day)	40 ± 12	38 ± 12	44 ± 15
TPOAb (IU/mL)	840 ± 325	901 ± 382	878 ± 298
TgAb (IU/mL)	815 ± 340	882 ± 404	860 ± 374
TSH (mIU/L)	2.9 ± 1.2	2.8 ± 1.1	3.0 ± 0.8
Free thyroxine	14.8 ± 2.7	14.6 ± 2.1	14.9± 2.8
Free triiodothyronine (pmol/L)	3.1 ± 0.8	3.1 ± 0.9	3.2 ± 0.9
Free triiodothyronine/free thyroxine ratio (pmol/L)	0.209 ± 0.047	0.212 ± 0.051	0.215 ± 0.043
25OHD (nmol/L)	110.5 ± 19.5	113.8 ± 15.9	115.1 ± 17.6
Jostel’s TSH index	3.1 ± 0.2	3.0 ± 0.2	3.1 ± 0.2
SPINA-GT (pmol/s)	2.19 ± 0.50	2.21 ± 0.46	2.17 ± 0.51
SPINA-GD (nmol/s)	19.40 ± 4.51	19.63 ± 4.20	19.88 ± 5.02
Estradiol (pmol/L)	286 ± 102	328 ± 115	298 ± 120
Testosterone (nmol/L)	1.10 ± 0.40 ^#&^	0.88 ± 0.38	0.84 ± 0.43
Prolactin (ng/mL)	18.0 ± 6.2	17.5 ± 7.4	16.9 ± 8.0
FSFI	26.52 ± 4.12	24.40 ± 4.62	25.25 ± 4.70
BDI-II	13.6 ± 4.1	14.2 ± 3.5	14.0 ± 4.2

Group A: adequate vitamin E intake; Group B: low vitamin E intake; Group C: high vitamin E intake. Values are expressed as mean ± standard deviation, unless noted otherwise. * *p* < 0.05 vs. group A; ^#^ *p* < 0.05 vs. group B; ^&^ *p* < 0.05 vs. group C.

**Table 2:** Sexual dysfunction and depressive symptoms of varying severity in reproductive-age women with euthyroid autoimmune thyroiditis before and after selenomethionine treatment, stratified by vitamin E status.

Variable	Group A	Group B	Group C
**FSFI score ≤ 26.55**			
At study entry	12 (50)	15 (62)	14 (56)
At study end	6 (25) *^#^	14 (58)	13 (52)
**Depressive symptoms**			
At study entry	12 (50)	13 (54)	14 (56)
At study end	5 (21) *^#^	13 (54)	12 (48)
**Mild symptoms**			
At study entry	12 (50)	12 (50)	13 (52)
At study end	5 (21) *^#^	12 (50)	11 (44)
**Moderate symptoms**			
At study entry	0 (0)	1 (4)	1 (4)
At study end	0 (0)	1 (4)	2 (8)
**Severe symptoms**			
At study entry	0 (0)	1 (4)	1 (4)
At study end	0 (0)	1 (4)	0 (0)

Group A: adequate vitamin E intake; Group B: low vitamin E intake; Group C: high vitamin E intake. Data are expressed as the number of patients, with percentages indicated in square brackets. * *p* < 0.05 vs. the corresponding values in groups B and C. ^#^ *p* < 0.05 vs. baseline values within the same group.

**Table 3:** Relationships between selenomethionine’s impact on biochemical variables and sexual function in the study population.

Correlated Variables		Group A	Group B	Group C
Δ FSFI score	Δ TPOAb	0.55 ^&^	0.40 ^#^	0.38 ^#^
Δ FSFI score	Δ TgAb	0.51 ^&^	0.36 ^#^	0.34 *
Δ Desire	Δ TPOAb	0.34 *	0.28 *	0.29 *
Δ Desire	Δ TgAb	0.31 *	0.26 *	0.26 *
Δ Desire	Δ Testosterone	0.49 ^&^	0.39 ^#^	0.40 ^#^
Δ Arousal	Δ TPOAb	0.35 ^#^	0.32 *	0.29 *
Δ Arousal	Δ TgAb	0.30 *	0.27 *	0.25 *
Δ Arousal	Δ Testosterone	0.47 ^&^	0.42 ^#^	0.43 ^#^
Δ Lubrication	Δ TPOAb	0.50 ^&^	0.35 ^#^	0.36 ^#^
Δ Lubrication	Δ TgAb	0.44 ^#^	0.34 *	0.29 *
Δ Lubrication	Estradiol	0.43 ^#^	0.46 ^#^	0.39 ^#^
Δ Orgasm	Δ TPOAb	0.30 *	0.29 *	0.32 *
Δ Orgasm	Δ TgAb	0.28 *	0.25 *	0.30 *
Δ Sexual satisfaction	Δ TPOAb	0.48 ^&^	0.41 ^#^	0.38 ^#^
Δ Sexual satisfaction	Δ TgAb	0.44 ^#^	0.39 ^#^	0.38 ^#^
Δ Pain	Δ TPOAb	0.46 ^#^	0.40 ^#^	0.41 ^#^
Δ Pain	Δ TgAb	0.41 ^#^	0.42 ^#^	0.44 ^#^

Group A: adequate vitamin E intake; Group B: low vitamin E intake; Group C: high vitamin E intake. Data are expressed as correlation coefficients (r values). * *p* < 0.05, ^#^ *p* < 0.01, ^&^ *p* < 0.001.

**Table 4:** Relationships between selenomethionine’s impact on sexual function and mean daily vitamin E intake during the study in women with normal (group A) and low (group B) intake.

Correlated Variable	Group A	Group B
Δ FSFI score	0.49 ^&^	0.52 ^&^
Δ Desire	0.44 ^#^	0.48 ^&^
Δ Arousal	0.40 ^#^	0.47 ^&^
Δ Lubrication	0.32 *	0.44 ^#^
Δ Orgasm	0.29 *	0.42 ^#^
Δ Sexual satisfaction	0.28 *	0.49 ^&^
Δ Pain	0.38 ^#^	0.46 ^#^

Data are expressed as correlation coefficients (r values). * *p* < 0.05, ^#^ *p* < 0.01, ^&^ *p* < 0.001.

**Table 5:** Relationships between selenomethionine’s impact on sexual function and depressive symptoms in the study population.

Correlated Variables		Group A	Group B	Group C
Δ BDI-II	Δ FSFI score	0.55 ^&^	0.48 ^&^	0.53 ^&^
Δ BDI-II	Δ Desire	0.52 ^&^	0.50 ^&^	0.48 ^&^
Δ BDI-II	Δ Arousal	0.49 ^&^	0.47 ^&^	0.47 ^#^
Δ BDI-II	Δ Lubrication	0.44 ^#^	0.35 *	0.34 *
Δ BDI-II	Δ Orgasm	0.39 ^#^	0.42 ^#^	0.42 ^#^
Δ BDI-II	Δ Sexual satisfaction	0.41 ^#^	0.34 *	0.29 *
Δ BDI-II	Δ Pain	0.40 ^#^	0.44 ^#^	0.35 *

Group A: adequate vitamin E intake; Group B: low vitamin E intake; Group C: high vitamin E intake. Data are expressed as correlation coefficients (r values). * *p* < 0.05, ^#^ *p* < 0.01, ^&^ *p* < 0.001.

**Table 6:** Multivariate linear regression analysis of sexual functioning.

Variable	Adjusted Partial R^2^ for Δ TPOAb	Adjusted Partial R^2^ for Mean Daily Vitamin E Intake ^$^
Δ FSFI score	0.402 ^&^	0.419 ^&^
Δ Desire	0.172 ^#^	0.410 ^&^
Δ Arousal	0.142 *	0.355 ^&^
Δ Lubrication	0.250 ^&^	0.231 ^#^
Δ Orgasm	0.259 ^&^	0.293 ^&^
Δ Sexual satisfaction	0.312 ^&^	0.285 ^&^
Δ Pain	0.286 ^&^	0.274 ^&^

Group A: adequate vitamin E intake; Group B: low vitamin E intake; Group C: high vitamin E intake. * *p* < 0.05, ^#^ *p* < 0.01, ^&^ *p* < 0.001. ^$^ only if Groups A and B were analyzed.

## Data Availability

The original contributions presented in this study are included in the article. Further inquiries can be directed to the corresponding author.
